# Increased performance uncertainty in children with ADHD? - Elevated post-imperative negative variation (PINV) over the ventrolateral prefrontal cortex

**DOI:** 10.1186/1744-9081-7-38

**Published:** 2011-08-25

**Authors:** Janina Werner, Matthias Weisbrod, Franz Resch, Veit Roessner, Stephan Bender

**Affiliations:** 1 Department of Child and Adolescent Psychiatry, University of Heidelberg, Blumenstraße 8, D-69115 Heidelberg, Germany; 2 Section for Experimental Psychopathology, Psychiatric Hospital, Voßstraße 4, D-69115 Heidelberg, University of Heidelberg, Germany; 3 SRH-Klinikum Karlsbad-Langensteinbach, Psychiatric Hospital, Guttmannstraße 1, D-76307 Karlsbad, Germany; 4 Department of Child and Adolescent Psychiatry, Medical School Carl Gustav Carus, Dresden University of Technology, Fetscherstraße 74, D-01307 Dresden, Germany; 5 Department of Child and Adolescent Psychiatry, University of Frankfurt, Deutschordenstraße 50, D-60528 Frankfurt am Main, Germany

## Abstract

**Background:**

We aimed to investigate the influences of attention deficit/hyperactivity disorder (ADHD) on response evaluation, as reflected by the postimperative negative variation (PINV), a slow event-related potential.

**Methods:**

We investigated PINV as an indicator of performance uncertainty in an audio-visual contingent negative variation (CNV) paradigm with an interstimulus interval of 3 seconds. A constant, unilateral, quick motor reaction with either the right or the left thumb was required after an auditory forewarned (S1) visual imperative stimulus (S2). We examined 18 ADHD patients (combined or hyperactive-impulsive subtype) aged between 8 and 14 years and an age-, sex and IQ-matched control group of 19 healthy subjects using 64-channel high-density EEG. A first run was recorded drug-free, a second one under methylphenidate (MPH) medication in the ADHD group.

**Results:**

We found a significantly increased negativity of the PINV-component over the ventrolateral prefrontal cortex in ADHD children compared to the healthy control group. PINV amplitude was influenced by movement side, most likely due to the slightly more difficult task when left hand responses were required. After the intake of MPH, PINV amplitudes of ADHD children normalized.

**Conclusions:**

We conclude that children with ADHD are likely to be more uncertain about the correctness of their performance and interpret the increased PINV as a hint towards compensatory mechanisms for a deficit in the evaluation of contingencies. Further studies are needed to assess the exact extent to which remainders of eye-movement related potentials contribute to PINV amplitude despite the correction for eye-artifacts.

## Background

Attention deficit/hyperactivity disorder (ADHD) is one of the most common [1] and at the same time still not completely pathophysiologically understood child psychiatric diagnoses.

Clear deficits in executive functions like planning, inhibition and evaluation of movement have been found. On the other hand, it is still controversially discussed to which extent motivational aspects and deficits in delay aversion are responsible for the development of ADHD-typical symptoms [2].

Certain event-related potentials have been discussed as markers for the disorder but previous studies have pointed out heterogeneous neurophysiological profiles in ADHD patients [3,4].

Recently, a decreased error related negativity (ERN) over the anterior cingulate cortex (ACC) has been interpreted as ADHD children's diminished capacity to monitor their error responses and their failure to predict the likelihood that an error occurs in a given context [5-7].

These findings suggest deficits in the children's cognitive processing of movement caused by diminished internal monitoring processes [8].

In the current study we chose the PINV (postimperative negative variation) component as another important marker of movement/action monitoring processes, representing the individual's uncertainty about the correctness of a given answer [9], aimed to further investigate the disorders' influence on self-monitoring and the establishment of stable contingencies between stimuli and the corresponding required responses [10].

Enhanced PINV amplitudes have been found in a number of studies, reflecting a lack of control over aversive events, an unexpected change in controllability [11,12] and representing contingency reappraisal [13].

Especially schizophrenic [14,15] and depressive [16] individuals show elevated PINV amplitudes, representing the uncertainty about the appropriate response [17].

PINV amplitude is sensitive to ambiguous contingencies and is thought to reflect an unexpected outcome, which causes performance uncertainty [18]. PINV usually shows a (pre-)frontal maximum, so generators in the prefrontal cortex have been postulated [18]. A topographic multi-channel analysis of PINV in ADHD has not been performed so far.

We hypothesized that

• ADHD children's contingency evaluation and their cognitive performance monitoring is disturbed, resulting in an increased PINV amplitude of ADHD children compared to age-matched healthy controls.

• Methylphenidate (MPH) has a positive influence on the cognitive evaluation represented by a normalized PINV-amplitude after MPH-intake in the ADHD group.

## Methods

### Subjects

We analyzed eighteen right-handed (Edinburgh Handedness Inventory; EHI; [19]; laterality quotient mean value ± standard deviation 94.9 ± 10.2) children between 8 and 14 years (13 males and 5 females, mean age ± standard deviation 11.5 ± 1.9 years, mean IQ ± standard deviation 110.5 ± 18.8) who met the criteria of a hyperactive-impulsive or combined subtype of ADHD according to the semi-structured interview for DSM, K-SADS [20].

All patients were recruited either in the Child and Adolescent Psychiatric Department of the University of Heidelberg or at a child psychiatrist's practice, were treated with multilayer-release or immediate-release MPH without other co-medication and suffered from no other psychiatric diseases. This includes that we assured that there were no neuropsychiatric disorders such as psychoses and autism or neurological diseases as epilepsy [21], migraine [22] and tic-disorder [10], which are thought to lead to specific changes in contingent negative variation (CNV) parameters.

An IQ below 80 (4-subtest short version of HAWIK III [23]), led to exclusion from the study.

As Quinn et al. [24] found no significant difference between the concentration of multilayer- and immediate-release MPH within the first four hours after the intake, we included children treated with both immediate and extended release MPH in our patient group.

Twelve out of 18 ADHD children were treated with extended release MPH with a mean dosage of 0.85 mg/kg body weight (0.25 to 1.29 mg/kg), the other six with immediate release MPH with a mean dosage of 0.4 mg/kg (0.15 to 0.74 mg/kg).

Nineteen right-handed (EHI laterality quotient 97.3 ± 5.2) healthy, age-, gender- and IQ-matched children and adolescents (14 males and 5 females, mean age 11.6 ± 2.1 years, mean IQ 117.4 ± 13.0), who took no psychoactive medication and did not suffer from any neurological or psychiatric symptoms, were recruited as control group at Heidelberg's elementary and secondary schools.

In both groups we screened for visual impairments (corrected visus ≥ 0.8).

All subjects and their parents provided written informed consent according to the Declaration of Helsinki and the study was approved by the local ethics committee.

### Task/recording/data pre-processing

We recorded a CNV paradigm, using an auditory warning stimulus S1 (1000 Hz, 90 dB, 50ms duration) and a visual imperative stimulus S2 (image of a white hand, pointing towards the side of the required button press, presented for 150 ms on a black screen). The interstimulus interval was 3 seconds, intertrial intervals varied randomly from 7 to 11 seconds.

Subjects were instructed to correctly respond as fast as possible when S2 occurred on the screen by pressing a button on the STIM response pad (Neuroscan Inc, TX, USA) with the thumb of either the right or the left hand (quick, unilateral motor answer).

40 trials per hand were recorded in a counterbalanced order across subjects. Two runs were recorded: In the control group both runs, T1 and T2, were drug-free. In the ADHD group, the first one (T1) was drug-free (after at least 24 hours after the last intake of MPH), the second one (T2) after 70 minutes after the intake of the individual used dose of MPH. In other studies the same experimental period of 70 minutes after the intake of MPH was chosen, so comparability is ensured.

Participants fixated a cross on a computer screen in order to minimize eye artifacts. Neuroscan Synamp Amplifiers (Neuroscan Inc., USA) were used to record continuous DC 64-channel EEG with a sampling rate of 250 Hz. An anti-aliasing filter was set at 70 Hz (low-pass). Surface Ag-AgCl sintered electrodes were fixed using an equidistant electrode cap (Easycap, FMS, Germany) and are named according to an extended international 10-20 system. The vertical and horizontal electrooculogram (EOG) was recorded by electrodes 1 cm next to the outer canthi and above/below the left eye.

Impedances were kept below 5 kΩ. Data were recorded against a reference near Cz and transformed offline to average reference. Recordings 1 s before S1 served as baseline. For the analysis of PINV, the EEG-signal was digitally filtered (30 Hz high cut-off), segmented into epochs of 7.5 s (1 s pre S1 to 3.5 s post S2), corrected automatically for DC-drifts by linear regression (Brain Vision Analyzer, Brain Products GmbH, Germany), and for eye movements and blinks (algorithm according to Gratton and Coles as implemented in Brain Vision Analyzer Version 1).

Artifacts were rejected automatically if the signal amplitude exceeded 150 mV. This procedure was confirmed by visual inspection; only artifact free trials entered further analysis. Bad channels were interpolated using nearest neighbours. Trials were rejected from further analysis if subjects responded with the wrong hand or after more than 3.5 s after S2.

Two ADHD patients and one control child (out of originally n = 20 children in both groups) had to be excluded from further evaluation due to recording errors or excessive artifact-prone data. N = 18 ADHD children and n = 19 controls were included for further statistical analysis.

### Data analysis/statistics

As a first step, planned comparisons for group differences between the PINV-amplitudes of unmedicated ADHD versus control children over the left and right ventrolateral prefrontal areas (pooled leads AF7, FP1, F9 and AF8, FP2, F10 during the time interval 2000 to 3000 ms after the imperative stimulus S2 in agreement with results of our previous study [18]) were examined for right and left hand button presses by four t-tests. The significance level was set to p = 0.05/4 = 0.0125 (Bonferroni correction).

Next, in order to assess the influence of medication on PINV topography in more detail, results were examined by multivariate analysis of variance (MANOVA), using the between subject factor GROUP (ADHD versus healthy controls) and the within subject factors SIDE of the response movement (left vs. right hand), HEMISPHERE (left vs. right VLPFC), RUN (T1 vs. T2) and ELECTRODES (AF7/8, FP1/2, F9/10) followed by simpler separate MANOVAs for left and right hand response CNV tasks.

Significant main effects or interactions in the MANOVA were subsequently further examined by post-hoc tests (Newman Keuls).

## Results

### 1. Group differences between drug-free ADHD patients and control children (medication-free first run)

#### 1.1 Behavioral data - reaction times

Mean reaction times (± standard deviation) were 317 ± 65 ms (right hand button presses) and 333 ± 84 ms (left hand button presses) for children with ADHD as well as 305 ± 63 ms (right hand button presses) and 320 ± 81 ms (left hand button presses) for healthy control children.

A repeated measurements ANOVA with the factors SIDE of the response movement and GROUP (ADHD versus healthy controls) revealed shorter reaction times for responses with the dominant right than with the left hand (F(1;35) = 5.9; p = 0.02). In contrast, GROUP had no significant main effect on reaction times (F(1;35) = 0.3; p > 0.59) and did not interact with response movement side either (F(1;35) = 0.0; p > 0.90).

#### 1.2 EEG data - PINV

Group differences of the PINV amplitudes over the ventrolateral prefrontal areas between unmedicated ADHD patients and the control group (T1) are presented for left (Table 1) and right hand response movements (Table 2). Mean values and standard deviations as well as the results of the four t-tests are shown.

**Table 1:** Group differences in PINV amplitude for left hand responses

*right hemisphere mean [μV] ± standard deviation*	AF8	FP2	F10	right VLPFC	*Difference right VLPFC ADHD vs CO*
**Control group (N = 19)**	-2.49 ± 6.72	-0.78 ± 5.21	-1.08 ± 5.90	***-1.45 ± 5.21***	***t = 2.73*** ***p = 0.0099***
**ADHD group (N = 18)**	-6.32 ± 5.63	-6.53 ± 7.66	-5.05 ± 6.20	***-5.97 ± 4.83***	
***left hemisphere ****mean [μV] ± standard deviation*	**AF7**	**FP1**	**F9**	**left VLPFC**	*Difference left VLPFC ADHD vs CO*
**Control group (N = 19)**	0.26 ±4.84	-0.34 ± 4.60	-0.23 ± 5.81	-0.10 ± 3.66	t = 1.06 p = 0.30
**ADHD group (N = 18)**	-0.70 ± 5.84	-4.49 ± 9.06	-0.60 ± 7.06	-1.93 ± 6.49	

PINV amplitudes (mean values and standard deviations) over the right and left ventrolateral prefrontal area (VLPFC) for unilateral response movement with the left hand. CO = healthy control group. Significant differences are presented in bold italics.

**Table 2:** Group differences in PINV amplitude for right hand responses

*right hemisphere mean [μV] ± standard deviation*	AF8	FP2	F10	right VLPFC	*Difference right VLPFC ADHD vs CO*
**Control group (N = 19)**	-3.01 ± 7.25	-3.44 ± 10.14	-3.00 ± 6.35	-3.15 ± 6.62	t = 0.84 p = 0.41
**ADHD group (N = 18)**	-4.76 ± 5.45	-5.42 ± 6.32	-4.02 ± 5.46	-4.73 ± 4.66	
***left hemisphere ****mean [μV] ± standard deviation*	**AF7**	**FP1**	**F9**	**left VLPFC**	*Difference left VLPFC ADHD vs CO*
**Control group (N = 19)**	-1.08 ± 5.83	-0.82 ± 5.20	-2.04 ± 3.79	-1.32 ± 3.83	t = 0.38 p = 0.71
**ADHD group (N = 18)**	-0.66 ± 5.01	-5.81 ± 9.04	0.55 ± 11.12	-1.97 ± 6.48	

PINV amplitudes (mean values and standard deviations) over the right and left ventrolateral prefrontal area for unilateral response movements with the right hand. CO = healthy control group.

**Figure 1:**
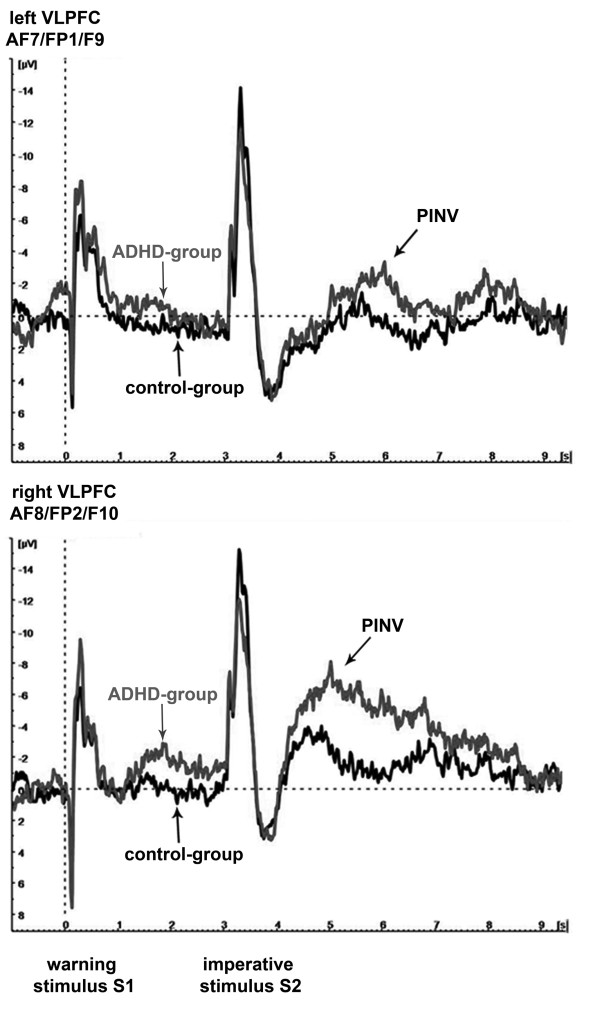
**PINV time course: ADHD versus healthy control children**. PINV time-course over the left (top) and the right (bottom) ventrolateral prefrontal cortex for unilateral response movement with the left thumb. The potentials of ADHD children are depicted in grey, those of the control-group in black. The drug-free first run T1 is shown. The vertical dashed line indicates the time when the auditory warning stimulus S1 occurred, the visual imperative stimulus S2 followed 3 s later.

We found significantly elevated PINV amplitudes over the right ventrolateral prefrontal cortex (VLPFC) in the ADHD group in comparison to the healthy control group, when the unilateral motor response was given by the left hand (p = 0.01; t = 2.7). For the ipsilateral, left prefrontal area there was no significant difference (p = 0.30, t = 1.1). Figure 1 shows the time course of the prefrontal PINV amplitudes separately for each hemisphere when the unilateral response movement is given with the left hand.

For the unilateral response movement with the right hand, there were no significant group differences (cf. Tables 1 and 2).

**Figure 2:**
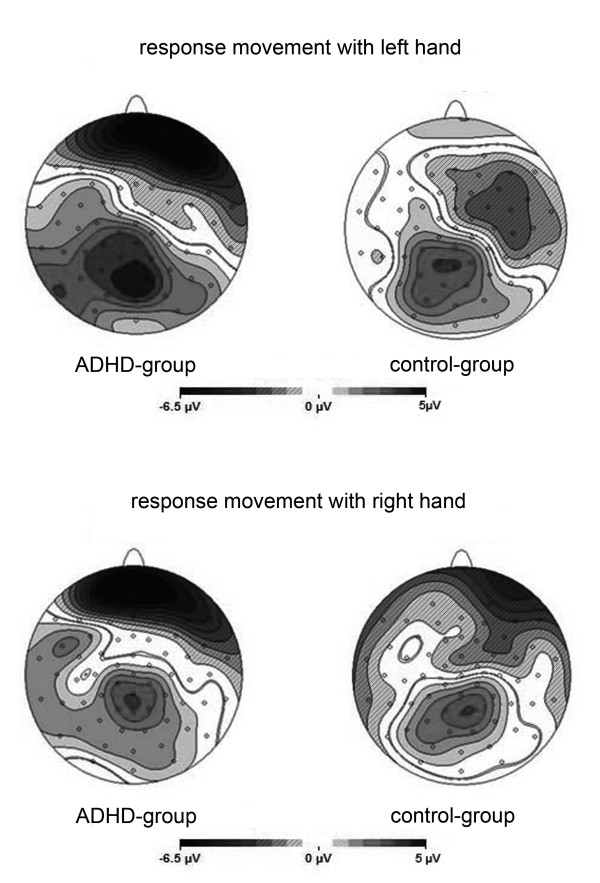
**PINV topography: ADHD versus healthy control children**. Comparison of the PINV topographies 2000 to 3000 ms after the imperative stimulus S2 in ADHD children versus healthy control children. The activation over the prefrontal cortex is displayed by different shades of grey. Current sinks (negative potential shifts) are presented striped and current sources (positive potential shifts) without stripes with a scale ranging from -6.5 μV to +5 μV.

The topographical analysis of the cortical activation 2000 to 3000 ms after the target stimulus S2 is shown in Figure 2 (reference-free current source density maps), illustrating the above-described group differences. Irrespective of the side of the response movement, a higher right-sided negativity over ventrolateral prefrontal areas during PINV is obvious, although the lateralization of the activation is noticeably weaker when the response movement is given by the right thumb.

### 2. Separation of PINV and eye movement and blink artifacts

**Figure 3:**
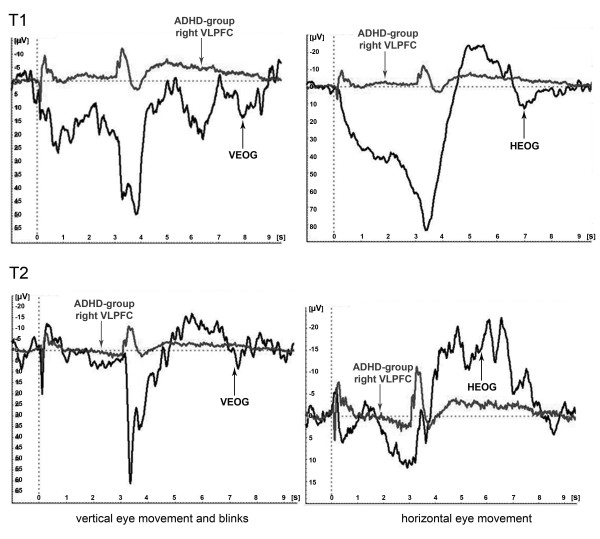
**Evoked EEG response (PINV) versus Electrooculogram (EOG)**. Comparison of the potential time course over the right ventrolateral prefrontal area in ADHD children versus their electrooculogram (group grand averages). The horizontal and vertical EOG is depicted in black, PINV amplitudes over the right ventrolateral prefrontal cortex (AF8/FP2/F10) in grey.

In addition to the eye movement correction, we performed a comparison between the time-course of the electrooculagram and the PINV amplitudes, which revealed an independent time course as shown in Figure 3.

Although quite a lot of blink or eye movement artifacts occurred during the PINV interval (which made a complete removal of all trials with blink artifacts impossible), the visual examination of the single trials also confirmed that the time-course of the potentials in the leads over the ventrolateral prefrontal areas could not be explained by remainders of insufficiently corrected eye artifacts, as the time-courses differed in single trials as well.

### 3. Medication effects, detailed topographic analysis and comparison of left and right hand response trials

**Figure 4:**
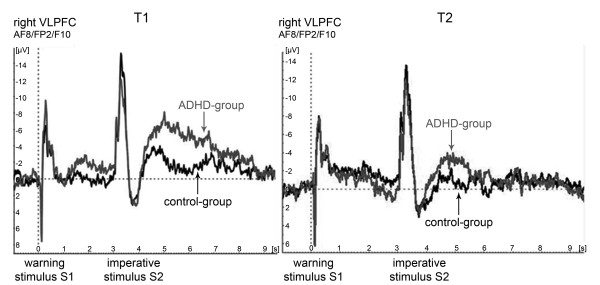
**Medication effects of methylphenidate**. PINV time course over the right ventrolateral prefrontal cortex (AF8/FP2/F10) at T1 and T2 for left hand button press trials. The potentials of the ADHD group are depicted in grey, those of the control-group in black.

During T2, about 70 minutes after the intake of MPH, the PINV amplitude in children with ADHD decreased to a normal level (Figure 4).

The overall MANOVA model (factors GROUP, SIDE of the response movement, HEMISPHERE, ELECTRODE and RUN) yielded an interaction between GROUP, SIDE of the response movement and ELECTRODE (F(2;34) = 6.0; p = 0.006. This interaction effect was further examined in separate MANOVAs for left and right hand response CNV tasks.

#### 3.1 Multivariate analysis of variance (MANOVA) for the unilateral response movement with the left hand with the factors diagnostic GROUP (ADHD versus healthy control children), HEMISPHERE (right versus left), ELECTRODES (AF7/8, FP1/2, F9/10) and RUN (T1 versus T2)

A main effect for the factor HEMISPHERE (F(1;35) = 12.8, p = 0.001) pointed towards higher PINV amplitudes over the right VLPFC (cf. Tables 1 and 2).

Furthermore, there was a trend towards an interaction between GROUP and RUN (F(1;35) = 3.8, p = 0.059). Newman Keuls post-hoc tests showed that this effect was due to a decrease in PINV amplitude after MPH intake in the ADHD group (p = 0.03), which could not be found in the healthy control group (p = 0.85).

An interaction of the factors HEMISPHERE x ELECTRODE (F(2;34) = 7.3, p = 0.002) indicated a different PINV topography for the left and right hemisphere. The strongest negativity was found at AF8 for the right hemisphere and at FP1 for the left hemisphere (see Tables 1 and 2).

Most important, there was an interaction of the factors (RUN x ELECTRODE x GROUP: F(2;34) = 10.1; p = 0.0004). Newman Keuls post-hoc tests showed that this effect has been identified as a consequence of a higher PINV amplitude especially in FP1/2 in T1 in the ADHD group. At FP1/2 during T1 there was a difference between the diagnostic groups (p = 0.005). For other combinations, e.g. at FP1/2 during T2 (p = 0.34) or at AF7/8 during T1 (p = 0.16) the level of significance was not reached for any group differences.

Table 3 gives a complete overview over the results of the MANOVA for the unilateral response movement with the left and the right hand; showing both significant and non-significant main effects and interactions.

**Table 3:** MANOVA for left and right hand responses

	MANOVA for left hand response		MANOVA for right hand response	
***Effect***	***F***	***p***	***F***	***p***

***Group***	2.42	0.13	1.59	0.22
***Run***	1.19	0.28	***6.54***	***0.02***
***Run x Group***	***3.80***	***0.06***	0.02	0.89
***Hemisphere***	***12.80***	***0.001***	***7.11***	***0.01***
***Hemisphere x Group***	0.98	0.33	0.16	0.69
***Electrode***	0.65	0.53	1.22	0.31
***Electrode x Group***	0.16	0.85	***3.89***	***0.03***
***Run x Hemisphere***	0.09	0.77	0.70	0.41
***Run x Hemisphere x Group***	1.11	0.30	0.09	0.77
***Run x Electrode***	0.52	0.60	1.34	0.27
***Run x Electrode x Group***	***10.08***	***0.00037***	0.48	0.63
***Hemisphere x Electrode***	***7.31***	***0.0023***	0.56	0.58
***Hemisphere x Electrode x Group***	0.95	0.40	2.15	0.13
***Run x Hemisphere x Electrode***	0.69	0.51	1.67	0.32
***Run x Hemisphere x Electrode x Group***	0.07	0.93	0.94	0.40

Multivariate analysis of variance of PINV amplitude after left and right hand responses examining the factors Hemisphere (left, right), Electrodes (AF7/8, FP1/2, F9/10), Run (T1,. T2) and Group (ADHD versus healthy controls). Significant results are presented in bold italics.

#### 3.2 MANOVA for the unilateral response movement with the right hand

A significant interaction of the factors GROUP x ELECTRODE (F(2;34) = 3.9, p = 0.03) irrespective of the run (T1/T2) indicated that the PINV amplitudes of children with ADHD were larger than those of healthy control children at FP1/FP2 but not other surrounding electrodes: Newman Keuls post-hoc tests showed a significant difference between the two diagnostic groups at the electrodes FP1/FP2 (p = 0.015), which did not exist for the other electrode positions, e.g. at F9/10 (p = 0.99).

A main effect for the factor RUN (F(1;35) = 6.54, p = 0.02) pointed towards lower amplitudes at T2.

Another main effect for the factor HEMISPHERE (F(1;35) = 7.11, p = 0.01) pointed towards higher amplitudes over the right hemisphere.

## Discussion

Our most important findings were

a) a significantly elevated negativity during the PINV over the VLPFC in unmedicated children with ADHD in comparison to healthy, age- and gender-matched subjects, especially when the unilateral response movement was given with the left hand. Longer reaction times indicated, that left hand responses represented the slightly more difficult task for our right-handed subjects. For right hand responses, there was also an elevated PINV amplitude in children with ADHD, but the PINV increase was more limited to leads Fp1/Fp2. Very easy tasks may decrease group differences. The differences in the healthy controls' PINV amplitudes between left and right hand responses (higher for the right handes) were not statistically significant and thus not further interpreted.

b) a normalization of the elevated negativity under MPH for elevated PINV amplitudes in the left hand response task, i.e. where the most pronounced group differences between unmedicated patients and healthy controls had been found.

### Elevated PINV amplitude as an expression of increased performance uncertainty in children with ADHD

Recent electrophysiological studies have suggested deficits in response monitoring and a diminished capacity to monitor error responses in children with ADHD, represented by decreased amplitudes of the ERN over the ACC, a region which is important for the discrimination between stimuli and the monitoring of actions and errors [25].

Our finding of a significantly elevated PINV amplitude over the VLPFC could be interpreted as a compensatory mechanism in the response monitoring process. Thus the deficits in error detection could by compensated by increased evaluation processing in other brain areas.

As the ACC and the VLPFC represent important parts of a monitoring network, responsible for the evaluation of the correctness of a given answer and the impairment of cognitive control in case of failure, the two cortex areas interact dynamically with each other and thus ensure the permanent self monitoring and adjustment of all target-oriented actions [26,27].

The VLPFC's role in particular is thought to be the processing of negative feedback in order to correct action with the objective of optimisation of performance; it is implicated in contingency detection and in the evaluation of stimuli [28,29].

An overall right-sided preponderance of PINV has been found also in previous studies [30] and points towards a preferential involvement of the right hemisphere in contingency evaluation. Apart from differences in task difficulty, this PINV lateralization could have also played a role for the more pronounced group differences in the left hand button press task.

Since the PINV appears in simple reaction time tasks, it seems to be a fundamental mechanism that plays a role in many goal-directed actions and is not limited to the two-stimulus situation of the CNV-paradigm.

The increased compensatory efforts for self-monitoring and contingency detection, represented by the enhanced PINV amplitude, may contribute to ADHD children's inability to concentrate on relevant stimuli in their environment.

Using functional magnetic resonance imaging (fMRI), a lower response in the right mesial prefrontal cortex was found during a stop task in hyperactive adolescents in comparison to healthy peers and it was concluded that ADHD is associated with subnormal activation of the prefrontal systems responsible for higher-order motor control [31]. From schizophrenia research, models that include both a compensatory (pre-)frontal hyperactivation or a (pre-)frontal hypoactivation depending on the difficulty of the examined task, are well established and may explain, why we found an *in*creased PINV over the right VLPFC while fMRI-studies highlight prefrontal processing *deficits *in more challenging tasks, despite clear differences between the deficits in subjects suffering from schizophrenia and subjects suffering from ADHD.

It has to be mentioned critically that in the inspection of the single trials we found some examples in which the potentials over the ventrolateral prefrontal area followed the time-course of the EOG before and after eye artifact correction. In general, PINV occurred independently from the EOG with a different time-course, as illustrated by our findings in the group grand average findings in Figure 3. After the elimination of all eye artifacts, unfortunately no reasonable number of trials remained for analysis. Therefore future studies must show the exact extent, to which the elevated amplitudes we found are contributed to by eye artifacts. ICA (independent component analysis)-based ocular correction approaches may yield additional information. However, our analyses showed that the described differences could not be explained sufficiently by eye artifacts in our sample.

### Interpretation of the normalization of PINV-amplitudes of ADHD patients after MPH intake

MPH, as an indirect dopaminergic agonist, could conceivably lead to an effect on error awareness, contingency evaluation and thus to modified PINV-amplitudes:

An increased ERN caused by stimulants could be found in adult patients [32], however, the intake of MPH had no effect on the ERN amplitude in another study by Groen et al. [6].

In other studies it was concluded that the inaccurate behaviour of ADHD children in conflict tasks might be related to reduced error-awareness and higher sensitivity to response conflict. The amelioration after the intake of MPH was interpreted as its positive influence on brain networks, enabling children with ADHD to allocate more attention to significant events [33].

MPH's influence on early error detection, however, seems to play a lesser role than the positive effect on subsequent processing steps. It has been concluded that the effect of MPH on self-monitoring processes is mediated rather by the noradrenergic than by the dopaminergic system [6].

However, no final statement can be made to which extent MPH has an influence on the amplitude of PINV:

Another important point, interpreting the approximation of the PINV-amplitudes in T2, could be learning effects due to the test repetition. In the second test run T2 the paradigm was already familiar to the children. They knew the stimulation and what reaction was expected and already practiced it in T1. Moreover, practice effects could differ between ADHD and healthy control children.

Accordingly, the increased performance uncertainty which we found in T1 could be decreased to a normal level (the uncertainty level of controls) in T2. The control group could not have shown any change between T1 and T2 due to a floor effect.

This interpretation would be consistent with the above-discussed theory of the PINV representing a deficient contingency evaluation.

In any case, the fact that differences in PINV amplitude were reduced and not produced by MPH shows that PINV differences were not due to acute medication effects of MPH (cf. Moll et al. [34]). Further studies may examine drug-naïve children.

## Conclusions

In the present study, we examined children with ADHD with regard to their slow movement related potentials in an audio-visual two-stimulus paradigm in comparison to age-matched, healthy controls.

We found a significant increase of the negative variation after the target stimulus S2 (PINV) over the right ventrolateral prefrontal cortex area in the ADHD group for left hand responses (the slightly more difficult task).

As elaborated above, the detected increase of the prefrontal PINV-amplitude can be interpreted as a deficit in contingency-evaluation representing ADHD children's higher uncertainty about the correctness of their own actions caused faulty monitoring processes.

The presented results can be used to better understand ADHD children's specific needs and incertitude. It may help to take another step into creating optimized learning conditions by reinsurance from the outside by immediate extremely clear feedback and drawing the child's attention to relevant stimuli to minimize the distraction by disturbed self-monitoring-processes.

## List of abbreviations

ACC: anterior cingulate cortex; ADHD: attention deficit/hyperactivity disorder; CNV: contingent negative variation; EOG: electrooculogram; ERN: error-related negativity; fMRI: functional magnetic resonance imaging; MPH: methylphenidate; PINV: postimperative negative variation; VLPFC: ventrolateral prefrontal cortex

## Competing interests

JW reports no competing interests.

MW has recently finished an investigator-initiated trial sponsored by Esparma and signed a contract concerning the development of neuropsychological diagnostic and training tools with Schuhfried.

FR reports no competing interests.

VR has received honoraries for lectures from Lilly, Medice, Novartis, Shire. He has been member of an advisory board of Lilly, Novartis. He has received research support from Novartis.

SB has received honoraries for lectures from Novartis and support for symposia by Shire.

## Authors' contributions

JW performed the EEG measurements, conducted large parts of data analysis and has drafted the manuscript.

MW and VR were involved in data interpretation and manuscript preparation.

FR was involved in fund raising, data interpretation and manuscript preparation.

SB planned the study design, supervised the data acquisition and analysis, and critically revised the manuscript.

All authors have read and approved the final manuscript.
